# Establishment and molecular characterization of decitabine‐resistant K562 cells

**DOI:** 10.1111/jcmm.14221

**Published:** 2019-02-22

**Authors:** Xiang‐Mei Wen, Ting‐Juan Zhang, Ji‐Chun Ma, Jing‐Dong Zhou, Zi‐Jun Xu, Xiao‐Wen Zhu, Qian Yuan, Run‐Bi Ji, Qin Chen, Zhao‐Qun Deng, Jiang Lin, Jun Qian

**Affiliations:** ^1^ Laboratory Center Affiliated People's Hospital of Jiangsu University Zhenjiang Jiangsu People's Republic of China; ^2^ Department of Hematology Affiliated People's Hospital of Jiangsu University Zhenjiang Jiangsu People's Republic of China; ^3^ The Key Lab of Precision Diagnosis and Treatment in Hematologic Malignancies of Zhenjiang City Zhenjiang Jiangsu P.R. China

**Keywords:** *DDX43*, decitabine, *H19*, K562, resistance

## Abstract

The clinical activity of decitabine (5‐aza‐2‐deoxycytidine, DAC), a hypomethylating agent, has been demonstrated in acute myeloid leukemia (AML) and myelodysplastic syndrome (MDS) patients. However, secondary resistance to this agent often occurs during treatment and leads to treatment failure. It is important to clarify the mechanisms underlying the resistance for improving the efficacy. In this study, by gradually increasing concentration after a continuous induction of DAC, we established the DAC‐resistant K562 cell line (K562/DAC) from its parental cell line K562. The proliferation and survival rate of K562/DAC was significantly increased, whereas the apoptosis rate was remarkably decreased than that of K562 after DAC treatment. In K562/DAC, a total of 108 genes were upregulated and 118 genes were downregulated by RNA‐Seq. In addition, we also observed aberrant expression of *DDX43/H19/miR‐186* axis (increased *DDX43*/*H19* and decreased *miR‐186*) in K562/DAC cells. Ectopic expression of *DDX43* in parental K562 cells rendered cells resistant to the DAC. Taken together, we successfully established DAC‐resistant K562 cell line which can serve as a good model for investigating DAC resistance mechanisms, and *DDX43/H19/miR‐186 *may be involved in DAC resistance in K562.

## 1 INTRODUCTION

DNA methylation is a major contributor to epigenetics involved in carcinogenesis especially in leukemogenesis. The balance is needed to be precisely maintained between DNA hypermethylation and hypomethylation, and dysregulation of the balance may give rise to human diseases.^1^ Abnormal DNA methylation changes, associated with DNA methyltransferases (*DNMT*s), are frequently observed in leukemia and supposedly contribute to disease occurrence and progression.^2^, ^3^ Therapy targeting DNA methylation modifiers has been regarded as a success in the treatment of hematopoietic malignancies.^4^, ^5^ Gene silencing caused by DNA hypermethylation can be reversed pharmacologically by prototypical *DNMT* inhibitors decitabine (5‐aza‐2‐deoxycytidine, DAC) and 5‐azacytidine (AZA), which have been recommended as one of the primary treatments for older acute myeloid leukemia (AML) and myelodysplastic syndrome (MDS) patients.^6^, ^7^, ^8^

The DAC is transported into the cell and then phosphorylated by deoxycytidine kinase (*DCK*) to the active metabolite 5‐aza‐dCTP, which incorporates into DNA during DNA replication to form a covalent complex with *DNMT*s, thereby inhibiting their activities followed by a reduction of DNA methylation, and consequently inducing anti‐leukemia effects.^9^ However, increasing clinical studies have found that resistance to such drug can develop during treatment and lead to treatment failure. Drug resistance was the major clinical obstacle to successful treatment of leukemia patients compared to patients with relatively sensitive cells. The clinical outcome of patients after failure with hypomethylating therapy was poor.^10^, ^11^ Insufficient incorporation into DNA was suggested to explain in vitro DAC resistance.^12^ It was reported that *DNMT3b* was upregulated in hypomethylating agent‐resistance cell lines.^13^ Also, high cytidine deaminase (CDA)/DCK ratio could be a mechanism of primary resistance to DAC in some patients.^14^

Nevertheless, the detailed mechanisms leading to DAC resistance still remains obscure. In this study, we induced K562 cell line for long periods of time using DAC to obtain the DAC‐resistant K562 cell line and investigated the potential mechanisms of DAC resistance.

## 2 MATERIALS AND METHODS

### 2.1 DAC‐resistant cell selection and cell culture

DAC‐resistant K562 cell line (K562/DAC) was established from its parental K562 cell line. The parental K562 cells were exposed continuously to gradually increasing concentrations of DAC. Original inducing DAC concentration was 2.5 µmol/L and then increased exponentially in each step till 320 µmol/L. The cells acquired resistance to DAC by a series of stepwise selections at last. Selected cells were cultured in DAC‐free medium prior to the experiment for at least 2 weeks. K562 and K562/DAC cells were incubated in Iscove's Modified Dulbecco's Medium (Wisent, Canada) containing 10% fetal bovine serum (FBS; ExCell Bio, Shanghai, China) and antibiotics at 37°C in a humidified, 5% CO_2_ atmosphere.

### 2.2 Morphology and measurement of drug sensitivity

An inverted light microscope (Nikon) and Wright‐Giemsa's compound stain were used to observe K562 and K562/DAC cells during the exponential phase. The nuclear to cytoplasm ratio of the cells was measured, which was the ratio of the diameter of the nucleus to the thickness of the cytoplasm on both sides.

K562 and K562/DAC cells were collected and placed in 6‐well plates at a density of 1 × 10^5^/mL with 2 mL medium. Fresh medium containing DAC at final concentration ranging from 0 to 2 µmol/L was added immediately, then fresh DAC was supplemented every 24 hours. After 96 hours, the surviving cells were calculated by trypan blue exclusion. The concentration of DAC required for 50% growth inhibition was scored as half maximal (50%) inhibitory concentration (IC50) value. The degree of resistance was evaluated by IC50 value. Each experiment was repeated three times. IC50 value of DAC was analyzed by the method of probit analysis in SPSS21.0 (SPSS Inc, USA).

### 2.3 Cell survival and proliferation assays

Cell viability of the K562 and K562/DAC cells were assessed. Briefly, cells were seeded in 6‐well plates at a density of 1 × 10^5^ cells/well with growth medium containing 0% FBS (cell survival assay) or 10% FBS (cell proliferation assay). DAC was added with the final concentration of 1 µmol/L for 96 hours. The results were presented from three independent experiments.

### 2.4 Cell apoptosis

To study cell apoptosis, cells were treated in 25 cm^2^ tissue culture flasks without FBS. Then cell apoptosis was evaluated with Annexin‐V‐FITC and propidium iodide (PI) double staining using an Annexin V apoptosis detection Kit (556547, Annexin V‐FITC Apoptosis Detection Kit I; BD, San Jose, CA, USA) according to the manufacturer's instructions, followed by flow cytometry analysis.

### 2.5 RNA‐Seq analysis

Total RNA was extracted from the cell samples by Trizol reagent (Invitrogen, Carlsbad, CA, USA) following the manufacturer's instructions. RNA was subjected to RNA‐Seq analysis by Beijing BerryGenomics Institute, China.

### 2.6 RQ‐PCR

cDNA was reverse transcribed from the RNA. Real‐time quantitative PCR (RQ‐PCR) was conducted to evaluate the mRNA and miRNA expression levels in the DAC resistant cells as previously described using the primer sets (Table S1).^15^, ^16^, ^17^, ^18^, ^19^

### 2.7 DNA isolation, chemical modification, RQ‐MSP and BSP

Genomic DNA isolation, chemical modification, real‐time quantitative methylation‐specific PCR (RQ‐MSP) and bisulfite sequencing PCR (BSP) were performed as our previous study.^15^, ^18^

### 2.8 *DDX43* stabled transfected K562 cell line

A lenti‐virus vector containing *DDX43* cDNA sequence was used to generate stable *DDX43*‐expressing K562 cell line. Then *DDX43* mRNA and protein were detected by real‐time quantitative PCR and western blot, respectively.^20^

### 2.9 Statistical analysis

All experiments were performed in triplicate (n ≥ 3) and the data were presented as mean ± SD. The Student's *t* test for independent samples was applied to define differences in the experiments. The differences of results were determined statistically significant if *P* was less than 0.05.

## 3 RESULTS

### 3.1 Establishment of DAC‐resistant cell line

**Figure 1:**
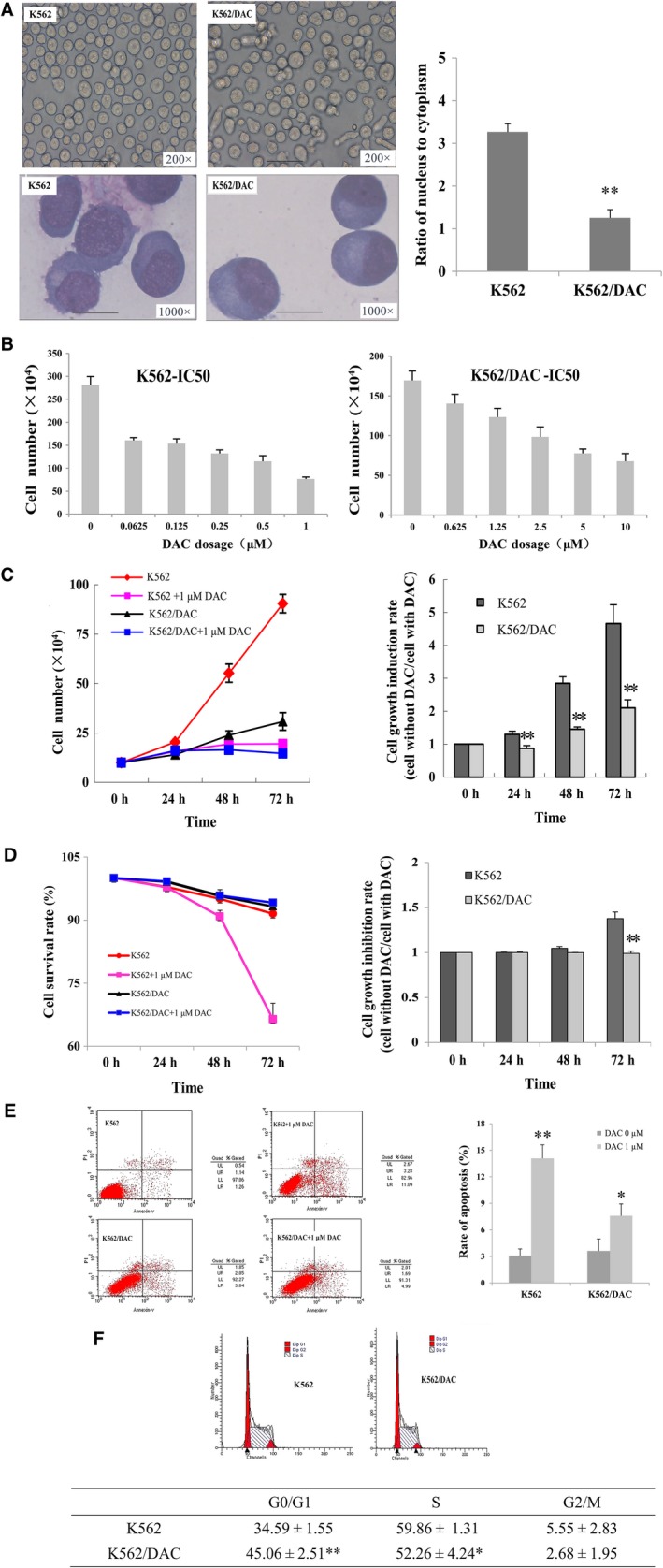
Establishment of DAC‐resistant K562 cell line. A, Morphological observation of K562 and K562/DAC cells (×200 magnification, bar = 50 μm; and ×1000 magnification, bar = 10 μm). K562/DAC cells had little cytologic atypia with smaller ratio of nucleus to cytoplasm. B, The concentrations of DAC required for 50% growth inhibition were scored as IC50 values. The IC50 values of K562 cell line and K562/DAC cell line to DAC were 0.26 ± 0.02 μmol/L and 3.16 ± 0.02 μmol/L, respectively. C, The proliferation of cells was analysed by cell counting with trypan blue dying in study group (with 1 μmol/L DAC) and control group (without DAC), then results were compared. D, Cells were maintained in serum‐free conditions. Surviving cells were harvested and counted for statistical analysis. E, Flow cytometry was performed after Annexin V‐FITC/PI staining. Results showed the percentage of apoptotic cells. F, Cell‐cycle distribution was measured by flow cytometry using PI, and the ratio of G0/G1 phase increased in K562/DAC cells. *P < 0.05, **P < 0.01 compared with control. Error bars indicate SD (n = 3)

Morphology differences between K562 and K562/DAC cells were surveyed using an inverted light microscope and Wright‐Giemsa's compound staining, and the results were shown in Figure 1A. K562 cells were homogeneous, yet K562/DAC cells were more irregular with little atypia under the light microscope. Wright‐Giemsa's compound staining showed that K562/DAC nucleus was more concentrated and the ratio of nucleus to cytoplasm became smaller compared with K562.

The IC50 value for DAC was 0.26 ± 0.02 μmol/L in K562 and 3.16 ± 0.02 μmol/L in K562/DAC (12‐fold increase compared to the parental cell line) (*P* < 0.05) (Figure 1B).

To further explore biological property of K562/DAC cells, we found that after treatment with DAC, K562/DAC cells had significantly higher proliferation and survival rates and lower apoptosis rate as compared to K562 cells (Figure 1C‐E). Meanwhile, the ratio of G0/G1 phase in K562/DAC increased (Figure 1F).

### 3.2 Gene expression alterations identified in K562/DAC cells

**Figure 2:**
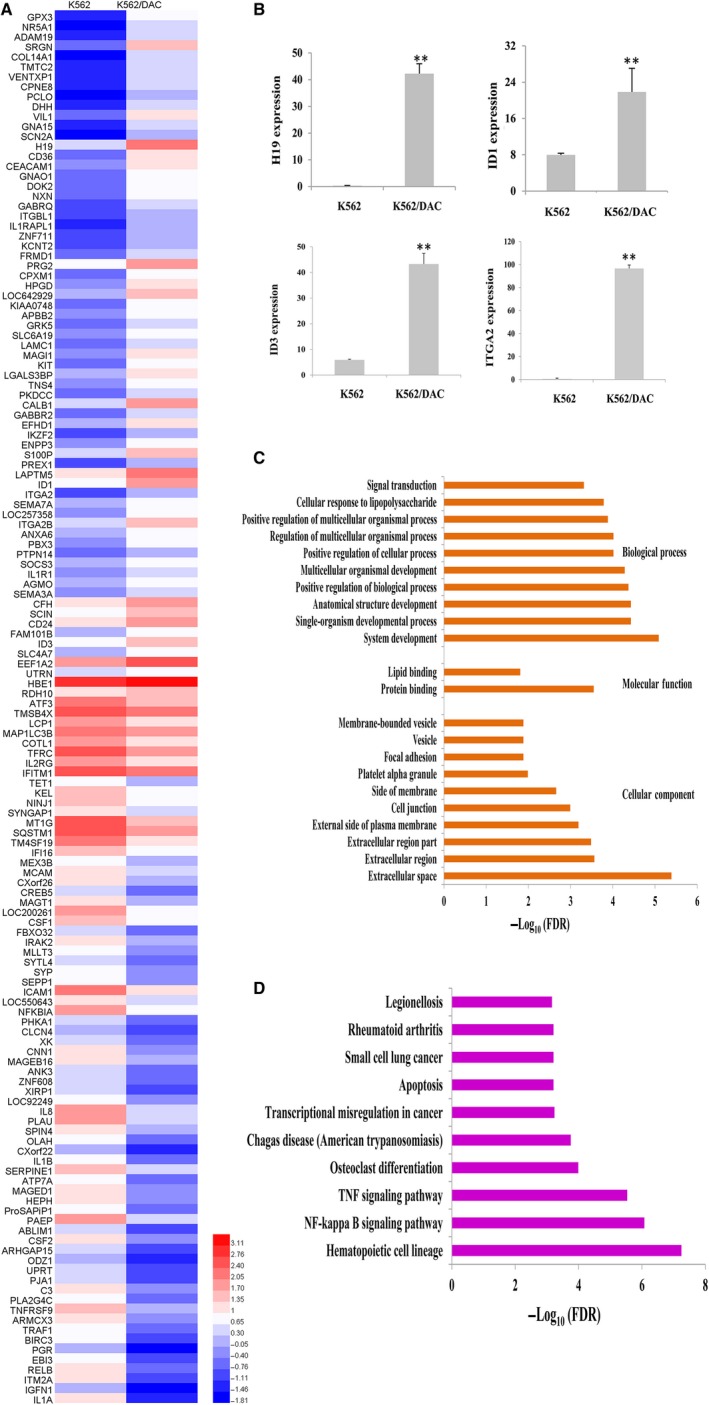
Analysis of the differentially expressed genes in K562/DAC cells. A, List of the top 140 differentially expressed mRNAs in K562/DAC cells compared to K562 cells. The color in each small boxes represents the expression level of the genes. Left lower panel: log values of reads per kilobase million in K562 and K562/DAC cells. B, Oncogene *H19*, *ID1*, *ID3* and *ITGA2* expression levels were confirmed with RQ‐PCR. Expression of *H19*, *ID1*, *ID3* and *ITGA2* were increased in the K562/DAC cells. **P < 0.01, compared with K562 cells. Error bars indicate SD (n = 3). C, GO enrichment analysis of differential genes. The genes were clustered according to the biological process, molecular function and cellular component. FDR: false discovery rates, false discovery rates <0.05. D, KEGG analysis of the top 10 significantly altered pathways in DAC‐resistant cells. FDR: false discovery rates, false discovery rates <0.05. The horizontal axis, −log_10_(FDR), denotes the significance of specific pathways in K562/DAC cells compared to K562 cells. GO, gene ontology; KEGG, Kyoto Encyclopaedia of Genes and Genomes

To recognize genes associated with DAC resistance, the candidate genes differentially expressed in K562 and K562/DAC cells were identified. RNA‐Seq analysis was used to screen the candidates (Figure 2A, Figure S1). Then RQ‐PCR was performed to validate the up‐regulated oncogene in K562/DAC cells. Four up‐regulated oncogenes in K562/DAC cells were validated by RQ‐PCR. The levels of *H19*, *ID1*, *ID3 *and *ITGA2* expressions dramatically increased in K562/DAC cells (Figure 2B). We also performed gene ontology (GO) enrichment analysis to classify differential genes into the categories of cellular component, molecular function and biological process, including extracellular space, protein binding and system development (Figure 2C). To gain deeper understanding the roles of these differentially expressed genes in K562/DAC, we further carried out KEGG pathway enrichment analysis. It was found that these genes were mostly enriched in hematopoietic cell lineage, NF‐kappa B signaling pathway and many other pathways (Figure 2D).

### 3.3 The role of *DDX43*/*H19*/*miR‐186* in DAC resistance

**Figure 3:**
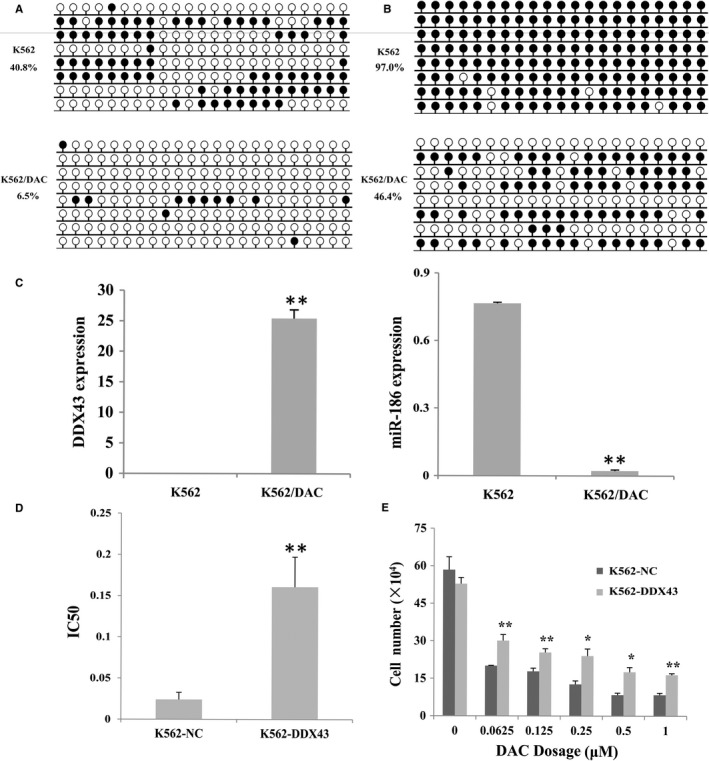
The role of DDX43/H19/miR‐186 in DAC resistance. A, *H19* methylation level detected by bisulfite sequencing in K562 and K562/DAC cells, respectively. B, Promoter methylation density of *DDX43* in K562 and K562/DAC cells. White cycle: unmethylated CpG dinucleotide; black cycle: methylated CpG dinucleotide. C, DDX43 expression was up‐regulated and inversely correlated with miR‐186 level in K562/DAC cell line. D, DAC resistance of K562 cells transfected with *DDX43 *(K562‐*DDX43*) and its control (K562‐NC) were tested. IC50 value increased in K562‐*DDX43*. E, The proliferation of K562‐*DDX43* was higher than that of control. *P < 0.05, **P < 0.01, compared with control. Error bars indicate SD (n = 3)

Our previous study had reported that overexpression of *DDX43 *in K562 cell line upregulated *H19* through demethylation.^20^ Also, *miR‐186* was found to target *DDX43*, and *miR‐186* was downregulated in *DDX43*‐transfected cells.^20^ Here, the density of *H19* and *DDX43* methylation was greatly decreased in K562/DAC cells (Figure 3A and B).

To further confirm the role of *DDX43* on the sensitivity of K562 cells to DAC, we performed RQ‐PCR to detect the expression of *DDX43* and *miR‐186*. *DDX43* expression level was increased and inversely correlated with *miR‐186* level in K562/DAC cell line (Figure 3C). The IC50 value for DAC was calculated both in K562 transfected with *DDX43* (K562‐*DDX43*) and the control (K562‐NC). The results showed that upregulation of *DDX43* enhanced DAC resistance of K562 cells compared with K562‐NC (IC50: 0.024 μmol/L vs 0.161 μmol/L; Figure 3D, *P* < 0.01). Transfection with *DDX43* could reduce sensitivity to DAC (Figure 3E).

## 4 DISCUSSION

The clinical outcome of patients after treatment failure with the DNA methylation inhibitors is poor in the clinics.^11^, ^21^ Therefore, it is important to illuminate the resistance mechanism and to overcome this problem. Drug‐resistant cell line models provide us with valuable in‐vitro tools in clarifying the mechanisms underlying clinical anticancer drug resistance. Cellular or molecular alterations can be detected between a drug‐resistant cell line and its drug‐sensitive counterpart. Furthermore, cell line models with acquired resistance play an additional and important role in discovering the action mechanism of new, developmental anticancer agents.^22^ Until now, it was reported that DAC‐resistant cells derived from HL‐60 and MOLM‐13 cells were investigated.^12^, ^13^ Herein, we developed a DAC‐resistant cell line by continuous exposure of K562 cell line to graded concentrations of the DAC. We also elucidated the phenotypic and molecular biology properties of our DAC resistance model. The IC50 value for DAC in K562/DAC cells was higher than that of K562 cells. Also, K562/DAC cells showed stronger tolerance after treatment with DAC. Establishment of DAC‐resistant cell line is not easy since half‐life time was 21 hours for DAC at physiologic media.^23^

In the study, we detected differentially expressed gene profiles to analyze whether a gene or signal pathway was involved using RNA‐Seq analysis. Our data presented distinct gene expression between parental K562 and DAC‐resistant cell line. The expression levels of oncogenes *H19*, *ID1*, *ID3* and *ITGA2* were upregulated in K562/DAC cells. The DNA methylation inhibitors have no specificity to particular regions due to its extensive demethylation.^24^ DNA methylation inhibitors not only up‐regulate tumor suppressor genes, but also activate some oncogenes, accordingly counteracting the anti‐tumor effect of tumor suppressor gene expression, and thus causing resistance in the tumor cells.^2^ The expression of some cancer‐related genes might be affected in the DAC‐resistant cell line. Some cancer‐related genes might play parts in the resistance by means of gene expression regulation. Meanwhile, GO and KEGG analysis illustrated that many fundamental genes and pathways were involved.

Enlighten by *DDX43/H19/miR‐186* axis facilitating tumorigenesis and CML progression, we then detected the expression level of *DDX43* and *miR‐186* in K562/DAC cells. Upregulated *DDX43* and downregulated *miR‐186* were identified in K562/DAC cells. Additionally, ectopic expression of *DDX43* in parental K562 cells induced cells resistant to DAC *DDX43*, initially found as a cancer/testis antigen, which is overexpressed in many solid tumors but absent in normal tissues except testis.^25^ *DDX43* was substantiated to stimulate oncogenic pathways responsible for cell proliferation.^26^ *DDX43* provided critical support to the progression of CML by enhancing cell survival and colony formation, and inhibiting cell apoptosis in vitro and in vivo.^20^ Moreover, DAC treatment in AML cell lines derepressed cancer/testis antigens localized on the X‐chromosome readily and transiently, which implied long‐term use of demethylated drugs may lead to genomic instability.^27^ Besides, *DDX43* could possibly serve as a new potential therapeutic target for recurrent colorectal cancer patients with chemoresistance.^28^ In selumetinib‐resistant uveal melanoma cell lines, *DDX43* was obviously overexpressed and mediated the induction of RAS protein expression and activity.^29^ In addition, *DDX43* inhibited IFN‐induced PML expression by promoting the suppressor of cytokine signaling 1 protein expression that inactivated the Janus kinase–signal transducers and activators of transcription signaling, consequently causing resistance of ABCB5_ malignant melanoma‐initiating cells (ABCB5_ MMICs) to IFNα.^30^ *H19* is a long chain non‐coding RNA with length of 2.3 kb. Several studies have reported that overexpression of *H19* was correlated with drug resistance in many tumors such as lung adenocarcinoma, ovarian cancer, human glioma, liver cancer.^31^, ^32^, ^33^, ^34^ Our previous study revealed that *H19* expression level, associated with its promoter methylation status, was significantly upregulated in CML patients involving in disease progression.^35^ Also, *H19* was identified to be upregulated by *DDX43* through demethylation related to CML progression.^20^ Among AML, *H19* overexpression correlated with poor chemotherapy response and shorter overall survival 36 Taken together, we deduced that *H19* may play a role in drug resistance during leukemogenesis. Thus, we detected the expression and methylation level of *H19* and *DDX43* in K562/DAC cells, and showed positive results. However, the relevance of methylation‐associated *DDX43*/*H19* with DAC resistance need to be further explored. The axis of *DDX43/H19/miR‐186* may be an attractive candidate for overcoming drug resistance in leukemia therapy.

In conclusion, a good in vitro model was successfully established, which can be used for elucidating the molecular mechanisms related to DAC resistance, and *DDX43/H19/miR‐186* axis may be associated with DAC resistance.

## CONFLICTS OF INTEREST

The authors stated that there are no conflicts of interest regarding the publication of this article. Research support played no role in the study design; in the collection, analysis, and interpretation of data; in the writing of the report; or in the decision to submit the report for publication.
