# Supplementary figures and images for: Establishment and molecular characterization of decitabine‐resistant K562 cells
Source: J Cell Mol Med. 2019 Feb 22;23(5):3317–24. doi: 10.1111/jcmm.14221 (PMC6484323; doi:10.1111/jcmm.14221)

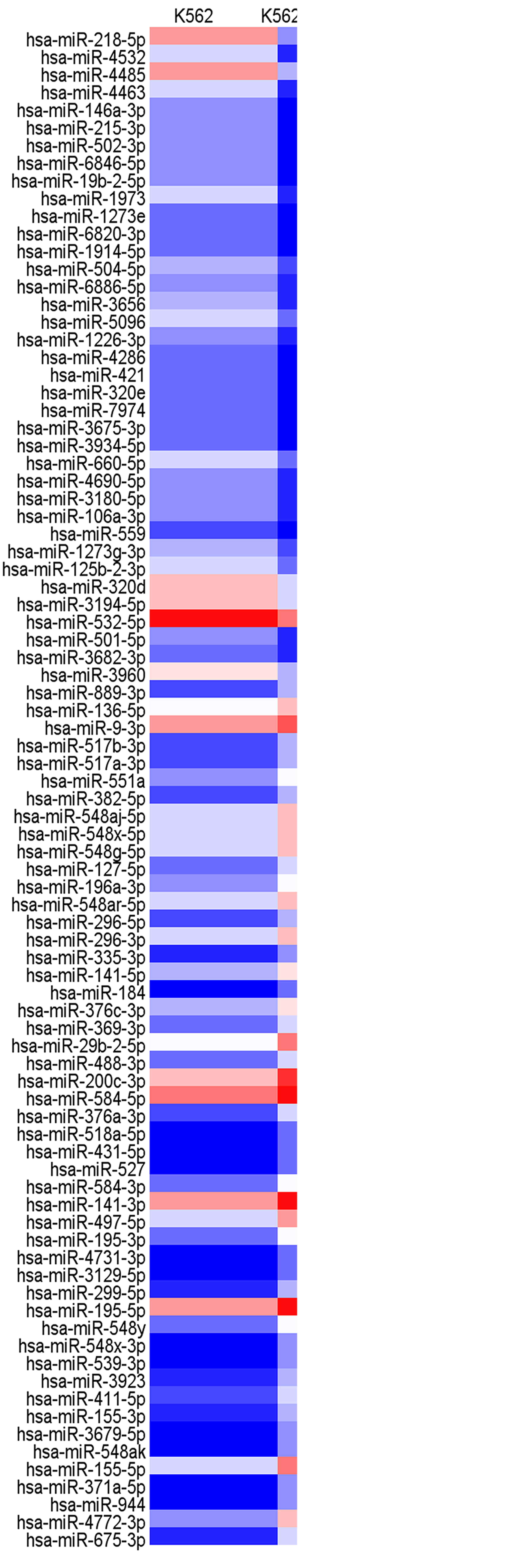

Supplement: Supplementary file 1 [file JCMM-23-3317-s001.tif]
