# Supplementary material for: Establishment and molecular characterization of decitabine‐resistant K562 cells
Source: J Cell Mol Med. 2019 Feb 22;23(5):3317–24. doi: 10.1111/jcmm.14221 (PMC6484323; doi:10.1111/jcmm.14221)
Supplement: Supplementary file 2 [file JCMM-23-3317-s002.doc]

**Supplementary Materials**

**Table 1．Primers used for RQ-PCR, BSP**

| Primers | Forward（5’→3’） | Reverse（5’→3’） | Predicted product size (bp) |
| --- | --- | --- | --- |
| RQ-PCR primers |  |  |  |
| *H19* | 5’-GGGTCAGACAGGGACATGG-3’ | 5’-GAGCGGTGAGGGCATACA-3’ | 354 |
| *ID1* | 5’-CTCAGCACCCTCAACGG-3’ | 5’-GATCGGTCTTGTTCTCCCTC-3’ | 199 |
| *ID3* | 5’-ACTCAGCTTAGCCAGGTGGA-3’ | 5’-AAGCTCCTTTTGTCGTTGGA-3’ | 274 |
| *ITGA2* | 5’-CTGGTGTTAGCGCTCAGTCA-3’ | 5’-TCGGTTCTCAGGAAAGCCAC-3’ | 186 |
| *DDX43* | 5’-CCTTTCAATGTTATCCTGAG-3’ | 5’-TATTCTTCAGATTGACGAAG-3’ | 432 |
| *miR-186* | 5’-CAAAGAATTCTCCTTTTGGGCT-3’ | miScript SYBR green PCR Kit |  |
| BSP primers |  |  |  |
| *H19*-B | 5’-TATGGGTATTTTTGGAGGTTTTTT-3’ | 5’-AAATCCCAAACCATAACACTAAAAC-3’ | 311 |
| *DDX43*-B | 5’-TTTTTTTTTGGAATAATGTTTTATTA-3’ | 5’-TAACCCCACCTATCCTACCCTAC-3’ | 307 |
